# Interactive effects of dietary energy levels with amino acid density on growth performance and optimal digestible Lys to energy ratio of male broiler chickens

**DOI:** 10.1016/j.psj.2024.104361

**Published:** 2024-09-26

**Authors:** Mehdi Toghyani, Shemil MacElline, Peter H. Selle, Sonia Y. Liu

**Affiliations:** ⁎ School of Life and Environmental Sciences, Faculty of Science, The University of Sydney, Camperdown, 2006, New South Wales, Australia; † Poultry Research Foundation, The University of Sydney, Camden, 2570, New South Wales, Australia; ‡ Sydney School of Veterinary Science, The University of Sydney, Sydney, New South Wales, 2006, Australia

**Keywords:** nutrient density, lysine to energy ratio, quadratic broken line model

## Abstract

A total of 2,400 day-old off-sex male Ross 308 chicks were used in a 4 × 3 factorial array (8 replicates with 25 birds per replicate) to determine the interactive effects of dietary metabolizable energy (**ME**) and amino acid (**AA**) densities on productive traits of broiler chickens reared to 42 d of age. The experimental factors were 4 ME levels (control, −50, −100, −150 kcal) and 3 digestible AA levels (control, +3.0 and +6.0%). Diets were fed for starter (0–10 d), grower (11−24 d), finisher (25–35 d), and withdrawal (36–42 d) phases, with consistent reduction of ME and increase in AA density for each phase. Overall, ME reduction did not compromise final body weight (BW; d 42). However, when ME was reduced by 50 kcal, only the diets with +6% AA density improved BW, resulting in an interaction between ME and AA levels (*P* < 0.05). Reducing ME and increasing AA densities independently increased total feed intake (*P* < 0.01). An interaction between ME and AA density on feed conversion ratio (**FCR**) was observed where increasing AA density at both +3.0 and +6.0% levels at control ME reduced FCR, but with reduced ME diets, FCR was reduced only at +6% AA density (*P* < 0.01). Quadratic broken line models estimated a digestible Lys to ME ratio (mg Lys/1,000 kcal ME) of 474 and 407 in starter and grower diets, respectively, to optimize both BW and FCR. However, in finisher phase a ratio of 363 could only be predicted for optimal FCR and in withdrawal a ratio of 349 to optimize BW. In summary, these results indicate that the interaction between ME and AA densities affects the productive traits of broiler chickens. Increasing AA density at control ME density improves FCR, while reduced ME diets require higher AA density to improve FCR. Quadratic broken line models suggest specific digestible Lys to ME ratios for optimizing BW and FCR across different phases of the production cycle.

## INTRODUCTION

Productive traits of broiler chickens such as rate of growth and feed conversion ratio have been exponentially improving because of genetic progress and precise nutrition (Aftab, 2019). Growth is a function of nutrient intake, and further increasing nutrient density can be expected to linearly improve feed efficiency, or more accurately, feed efficiency corrected for body weight. However, broiler chickens’ responses to diet nutrient density, particularly the most expensive and key nutrients: metabolizable energy (**ME**) and amino acids (**AA**), are curvilinear, and exhibit a phenomenon known as diminishing returns (Alhotan, 2021; Gous, 2007). Optimizing the balance between ME intake and expenditure is fundamental to the formulation of broiler chicken diets and levels of other dietary nutrients for efficient conversion of feed into muscle mass (Classen, 2017).

At constant high intakes of protein and other nutrients, an increasing supply of ME leads to a steady rise in protein deposition in tissues, up to a point where further ME provision primarily contributes to excessive body fat accumulation. This surplus fat typically correlates with poorer FCR (De Lange and Swanson, 2006; Lamot et al., 2019). The cut-off points where the ME supply is sufficient to support maximum protein accretion would, simultaneously, maximize efficiency of feed utilization (Campbell and Taverner, 1988). Although the exact level of dietary ME where the protein accretion hits its maximum is not known, evidence from recent studies suggest that the optimum level of ME is lower than what is provided in most commercial circumstances. (Chang et al., 2016; Sharma et al., 2018). More recent studies suggest that modern broiler chickens are more responsive to diet AA density than they are to ME density (Macelline et al., 2023). It has been reported that optimal dietary AA density is somewhere between 100 and 120% of breeder recommendations (Maharjan et al., 2020). Eventually, the 2 biggest broiler breeder suppliers revised their recommended specifications for dietary ME and AA density in late 2022, with the ME being reduced and the AA density being increased compared to their previous guidelines (Aviagen, 2022; Cobb-Vantress, 2022).

The current trial was conducted to determine the interactive effect of diet AA density with dietary ME levels against the latest Ross 308 recommendation (Aviagen, 2022) on growth performance and optimal digestible lysine to ME ratios for different growth periods of male broiler chickens raised to 42 d of age.

## MATERIAL AND METHODS

### Birds and Experimental Design

All the experimental protocols and procedures for the present study were reviewed and approved by the University of Sydney Animal Ethic Committee (protocol number AEC2022/2185). A total of 2,400 day-old off-sex male Ross 308 chicks was obtained from a commercial hatchery (Goulburn, NSW 2580). Upon arrival, birds were group weighed and assigned into their respective treatments into 96 floor pens. Each treatment was replicated 8 times with 25 birds per replicate. The feeding study consisted of 12 dietary treatments designed as a 4 × 3 factorial arrangement, which included 4 levels of dietary energy (control, −50, −100 and −150 kcal/kg) and 3 levels of amino acid densities (control, +3.0% and +6.0%) for each phase of the study. The diets were formulated to Ross 308 nutrients specification (Aviagen, 2022) and the reduction of energy and increase in amino acid density was applied to the base levels recommended by the breeder.

Prior to diet formulation, representative subsamples of wheat, soybean meal, meat and bone meal, canola meal and canola seed were analyzed by near-infrared spectroscopy to predict proximate analysis, digestible amino acid concentrations, and metabolizable energy (**ME**) using AMINONIR®PROX, AMINONIR®NIR, and AMINONIR® NRG (Evonik Nutrition & Care, Hanua, DE), respectively.

Diets were based on wheat (11.5 % CP; ME 3180), soybean meal (46.0 % CP; ME 2400), meat and bone meal (47.0 % CP; ME 2000), solvent canola meal (37.5 % CP; ME 1980) and canola seed (21.0 % CP; ME 4500), without any inorganic phosphate sources. A phytase dose of 2000 FYT/kg (Ronozyme HiPhorius 10) was included across all the diets with a matrix of 0.20 % Ca, 0.18% available P, 0.02% Na, 30 kcal/kg ME uplift and 50% of the manufacturer recommended matrix for amino acids. The diets were balanced for the essential amino acids including Lys, Met + Cys, Thr, Trp, Arg, Ile and Val by applying the ideal amino acid ratios recommended by the primary breeder (Aviagen, 2022). There was neither a cap nor a minimum set for dietary crude protein. Diets were formulated using least cost formulation. Ingredients and synthetic amino acids including Lys, Met, Thr, Arg, Ile, and Val were offered to the diets at market prices during the time of the study (Table 1, Table 2, Table 3, Table 4).

**Table 1:** Ingredients composition and key nutrient profiles of the starter diets (0–10 d).

Ingredients (%)	Control ME (2,975)			ME – 50 kcal (2,925)			ME – 100 kcal (2,875)			ME – 150 Kcal (2,825)		
	CAA^1^	MAA	HAA	CAA	MAA	HAA	CAA	MAA	HAA	CAA	MAA	HAA
Wheat 11.5%	57.78	55.84	53.73	59.03	56.99	54.94	60.48	58.19	56.13	61.36	59.22	57.13
Soybean Meal 46.0%	29.75	31.50	33.35	29.50	31.30	33.15	29.55	31.10	32.90	29.20	30.90	32.80
Meat meal 47 %	3.55	3.45	3.35	3.55	3.45	3.35	3.55	3.45	3.35	3.50	3.40	3.30
Canola Seeds	3.00	3.00	3.00	3.00	3.00	3.00	2.25	3.00	3.00	-	0.75	1.50
Canola Meal 37.5%	2.00	2.00	2.00	2.00	2.00	2.00	2.00	2.00	2.00	3.75	3.50	3.00
Canola Oil	1.750	2.000	2.300	0.750	1.050	1.300	-	0.050	0.350	-	-	-
Limestone	0.557	0.571	0.586	0.560	0.575	0.589	0.565	0.578	0.592	0.573	0.586	0.599
DL-Methionine	0.335	0.355	0.375	0.335	0.350	0.370	0.335	0.350	0.370	0.330	0.345	0.365
Lysine-HCl	0.330	0.330	0.335	0.330	0.335	0.335	0.335	0.335	0.340	0.340	0.340	0.340
Sodium Bicarbonate	0.265	0.265	0.270	0.270	0.270	0.270	0.270	0.270	0.270	0.275	0.275	0.275
Vit/Min Premix^2^	0.200	0.200	0.200	0.200	0.200	0.200	0.200	0.200	0.200	0.200	0.200	0.200
Salt	0.165	0.165	0.165	0.160	0.160	0.160	0.160	0.160	0.160	0.160	0.160	0.160
L-Threonine	0.150	0.155	0.160	0.150	0.155	0.160	0.150	0.155	0.160	0.150	0.155	0.160
Choline Chloride	0.080	0.080	0.080	0.080	0.080	0.080	0.080	0.080	0.080	0.080	0.080	0.080
L-Valine	0.030	0.036	0.042	0.028	0.034	0.040	0.028	0.033	0.038	0.027	0.032	0.038
Xylanase WX 2000	0.010	0.010	0.010	0.010	0.010	0.010	0.010	0.010	0.010	0.010	0.010	0.010
HiPhorius 10G 200 g	0.020	0.020	0.020	0.020	0.020	0.020	0.020	0.020	0.020	0.020	0.020	0.020
L-Isoleucine	0.014	0.016	0.018	0.013	0.015	0.017	0.012	0.014	0.016	0.012	0.014	0.016
Price AUD/T	$693.3	$708.7	$725.8	$671.5	$688.0	$703.9	$651.8	$666.4	$683.2	$637.8	$651.9	$667.0
Nutrients^3^												
AME Kcal/kg	2975	2975	2975	2925	2925	2925	2875	2875	2875	2825	2825	2825
Crude protein %	24.1	25.2	25.3	24.1	24.8	24.9	24.6	24.7	25.2	24.5	25.2	25.3
Crude fat %	4.71	5.02	5.22	4.01	4.22	4.28	3.02	3.25	3.42	2.11	2.31	2.39
Strach %	41.1	40.5	38.8	41.7	41.0	39.6	42.1	41.3	40.4	43.1	41.8	41.1
Total Lys %	1.34	1.41	1.44	1.37	1.39	1.44	1.40	1.44	1.45	1.38	1.41	1.45
Dig Lys %	1.32	1.36	1.40	1.32	1.36	1.40	1.32	1.36	1.40	1.32	1.36	1.40

1 CAA: control AA density, MAA: medium AA density (+3.0%), HAA: high AA density (+6.0%).

2 Vitamin concentrate supplied per kilogram of diet: retinol, 12000 IU; cholecalciferol, 5000 IU; tocopheryl acetate, 75 mg, menadione, 3 mg; thiamine, 3 mg; riboflavin, 8 mg; niacin, 55 mg; pantothenate, 13 mg; pyridoxine, 5 mg; folate, 2 mg; cyanocobalamin, 16 μg; biotin, 200 μg; cereal-based carrier, 149 mg; mineral oil, 2.5 mg. Trace mineral concentrate supplied per kilogram of diet: Cu (sulphate), 16 mg; Fe (sulphate), 40 mg; I (iodide), 1.25 mg; Se (selenate), 0.3 mg; Mn (sulphate and oxide), 120 mg; Zn (sulphate and oxide), 100 mg; cereal-based carrier, 128 mg; mineral oil, 3.75 mg.

3 Crude protein, crude fat, starch, and total lysine are determined values.

**Table 2:** Ingredients composition and key nutrient profiles of the grower diets (10–24 d).

Ingredients (%)	Control ME (3,050)			ME – 50 kcal (3,000)			ME – 100 kcal (2,950)			ME – 150 Kcal (2,900)		
	CAA^1^	MAA	HAA	CAA	MAA	HAA	CAA	MAA	HAA	CAA	MAA	HAA
Wheat 11.5%	60.98	58.87	57.34	62.12	60.12	58.59	63.27	61.27	59.69	64.84	62.66	60.83
Soybean Meal 46.0%	24.90	26.70	28.10	24.70	26.50	27.85	24.50	26.30	27.70	24.90	26.40	27.50
Canola Seeds	4.00	4.00	4.00	4.00	4.00	4.00	4.00	4.00	4.00	2.50	3.25	4.00
Canola Meal 37.5%	3.00	3.00	3.00	3.00	3.00	3.00	3.00	3.00	3.00	3.00	3.00	3.00
Canola Oil	2.40	2.70	2.90	1.45	1.70	1.90	0.50	0.75	0.95	-	-	-
Meat Meal 47 %	2.00	1.95	1.85	2.00	1.90	1.85	2.00	1.90	1.85	2.00	1.90	1.85
Limestone	0.76	0.78	0.79	0.77	0.78	0.79	0.77	0.79	0.80	0.78	0.79	0.80
Titanium Dioxide	0.50	0.50	0.50	0.50	0.50	0.50	0.50	0.50	0.50	0.50	0.50	0.50
Lysine-HCl	0.290	0.295	0.295	0.295	0.295	0.295	0.295	0.300	0.300	0.300	0.300	0.300
Sodium Bicarbonate	0.290	0.290	0.290	0.295	0.295	0.295	0.295	0.295	0.295	0.300	0.300	0.300
Dl-Methionine	0.280	0.300	0.315	0.280	0.300	0.315	0.275	0.295	0.310	0.280	0.295	0.310
Vit/Min Premix^2^	0.200	0.200	0.200	0.200	0.200	0.200	0.200	0.200	0.200	0.200	0.200	0.200
Salt	0.165	0.170	0.170	0.165	0.165	0.165	0.165	0.165	0.165	0.165	0.165	0.165
L-Threonine	0.110	0.120	0.125	0.110	0.120	0.120	0.110	0.115	0.120	0.115	0.120	0.120
Choline Chloride	0.070	0.070	0.070	0.070	0.070	0.070	0.070	0.070	0.070	0.070	0.070	0.070
Xylanase WX 2000	0.010	0.010	0.010	0.010	0.010	0.010	0.010	0.010	0.010	0.010	0.010	0.010
HiPhorius 10G 200 g	0.020	0.020	0.020	0.020	0.020	0.020	0.020	0.020	0.020	0.020	0.020	0.020
L-Valine	0.010	0.015	0.019	0.010	0.013	0.018	0.010	0.012	0.016	0.010	0.011	0.015
Price AUD/T	$671.5	$688.4	$700.4	$651.2	$666.6	$678.4	$630.6	$645.9	$658.1	$613.9	$626.7	$637.6
Nutrients^3^												
AME Kcal/kg	3050	3050	3050	3000	3000	3000	2950	2950	2950	2900	2900	2900
Crude protein %	22.2	23.3	23.6	22.4	22.8	23.7	22.7	23.5	23.4	22.4	23.6	23.7
Crude fat %	5.85	5.89	5.97	4.55	4.95	5.21	3.88	4.15	4.19	2.78	3.12	3.25
Strach %	41.5	41.1	40.7	42.8	42.6	41.3	42.8	42.5	40.8	43.7	43.7	42.3
Total Lys %	1.22	1.31	1.35	1.26	1.32	1.33	1.30	1.32	1.38	1.26	1.22	1.36
Dig Lys %	1.18	1.22	1.25	1.18	1.22	1.25	1.18	1.22	1.25	1.18	1.22	1.25

1 CAA: control AA density, MAA: medium AA density (+3.0%), HAA: high AA density (+6.0%).

2 Vitamin concentrate supplied per kilogram of diet: retinol, 12000 IU; cholecalciferol, 5000 IU; tocopheryl acetate, 75 mg, menadione, 3 mg; thiamine, 3 mg; riboflavin, 8 mg; niacin, 55 mg; pantothenate, 13 mg; pyridoxine, 5 mg; folate, 2 mg; cyanocobalamin, 16 μg; biotin, 200 μg; cereal-based carrier, 149 mg; mineral oil, 2.5 mg. Trace mineral concentrate supplied per kilogram of diet: Cu (sulphate), 16 mg; Fe (sulphate), 40 mg; I (iodide), 1.25 mg; Se (selenate), 0.3 mg; Mn (sulphate and oxide), 120 mg; Zn (sulphate and oxide), 100 mg; cereal-based carrier, 128 mg; mineral oil, 3.75 mg.

3 Crude protein, crude fat, starch, and total lysine are determined values.

### Data Collection and Analysis

Diets were steam-pelleted at a conditioning temperature of 80°C for 14 seconds. The pellet mill was equipped with a die ring with 4.0 mm holes and 38 mm thickness. The starter diets were further crumbled to maximize feed intake. All the diets were offered *ad libitum*. Pellet durability index (**PDI**) of all diets were tested, in triplicate, using the NHP 200 New Holman Automatic Pellet Tester (TekPro Ltd, Norfolk, UK).

Birds were weighed on a pen basis on d 0, 10, 24, 35 and 42 to determine body weights (**BW**) and calculate body weight gain (**BWG**). Feed intake (**FI**) was measured in similar intervals and used to calculate feed conversion ratio (**FCR**) for each phase. Mortality was recorded daily, and dead bird's BW was used to correct FCR values. On d 42, bird BW in treatment one (control ME and control AA densities) was used to calculate BW corrected FCR (FCRc) as there were treatment-associated differences in body weight. This correction was achieved by considering a 50 g difference in body weight was equivalent to 1 point (0.01) in FCR.

Diet prices, feed intake and feed conversion ratio data were used to calculate feed cost per kg of live body weight for each phase and the entire production period of 42 d. The average growth rate (g/b/day) was calculated for each treatment and used to calculate average age to 2.5 kg of live bodyweight.

Amino acid concentrations in the diets were determined by 24-h liquid hydrolysis at 110°C in 6 M HCl and then AA were analyzed using the Waters AccQTag Ultra chemistry (Waters) on a Waters Acquity UPLC (Milford, MA). The analyzed total amino acid concentrations in diets for phase I and phase II of the study are presented in Tables 3 and 5, respectively. The nitrogen contents of raw ingredients and diet samples were determined on a 0.25-g sample in a combustion analyzer (FP-2000 N analyzer; LECO, St Joseph, MI) using EDTA as a calibration standard, with CP being calculated by multiplying percentage N by a correction factor (6.25).

**Table 3:** Ingredients composition and key nutrient profiles of the finisher diets (24–35 d**)**.

Ingredients (%)	Control ME (3,100)			ME – 50 kcal (3,050)			ME – 100 kcal (3,000)			ME – 150 Kcal (2,950)		
	CAA^1^	MAA	HAA	CAA	MAA	HAA	CAA	MAA	HAA	CAA	MAA	HAA
Wheat 11.5%	65.33	63.56	61.78	66.43	64.90	62.98	67.62	66.09	64.27	69.55	67.92	65.91
Soybean Meal 46.2%	20.40	21.95	23.60	20.25	21.65	23.35	20.05	21.40	23.05	20.75	21.90	23.25
Canola Seeds	5.00	5.00	5.00	5.00	5.00	5.00	5.00	5.00	5.00	2.50	3.00	3.75
Canola Meal 37.5%	4.00	4.00	4.00	4.00	4.00	4.00	4.00	4.00	4.00	4.00	4.00	4.00
Canola Oil	2.10	2.35	2.55	1.15	1.35	1.60	0.15	0.40	0.60	-	-	-
Meat Meal 47 %	1.00	0.95	0.85	1.00	0.90	0.85	1.00	0.90	0.85	1.00	0.95	0.85
Limestone	0.81	0.83	0.84	0.82	0.83	0.84	0.82	0.83	0.85	0.83	0.84	0.85
Sodium Bicarbonate	0.285	0.285	0.280	0.285	0.285	0.280	0.290	0.290	0.285	0.295	0.295	0.290
Lysine-HCl	0.280	0.275	0.270	0.280	0.280	0.275	0.285	0.285	0.280	0.290	0.290	0.280
DL-Methionine	0.240	0.255	0.270	0.240	0.255	0.270	0.235	0.250	0.265	0.240	0.255	0.265
Vit/Min Premix^2^	0.200	0.200	0.200	0.200	0.200	0.200	0.200	0.200	0.200	0.200	0.200	0.200
Salt	0.160	0.160	0.165	0.160	0.160	0.160	0.155	0.155	0.160	0.155	0.155	0.160
L-Threonine	0.090	0.090	0.095	0.090	0.095	0.095	0.090	0.095	0.095	0.090	0.095	0.095
Choline Chloride	0.060	0.060	0.060	0.060	0.060	0.060	0.060	0.060	0.060	0.060	0.060	0.060
Xylanase WX 2000	0.010	0.010	0.010	0.010	0.010	0.010	0.010	0.010	0.010	0.010	0.010	0.010
HiPhorius 10G 200 g	0.020	0.020	0.020	0.020	0.020	0.020	0.020	0.020	0.020	0.020	0.020	0.020
Price AUD/T	$642.8	$656.2	$669.0	$622.6	$634.3	$648.5	$601.1	$613.6	$626.5	$586.8	$596.5	$607.7
Nutrients^3^												
AME Kcal/kg	3100	3100	3100	3050	3050	3050	3000	3000	3000	2950	2950	2950
Crude protein %	20.2	20.8	21.6	21.3	21.6	21.8	21.3	21.9	22.5	21.4	21.7	22.7
Crude fat %	5.45	5.90	6.21	4.55	5.11	5.32	3.95	3.94	4.45	3.11	3.15	3.45
Strach %	45.4	44.1	42.8	45.7	43.3	43.7	45.9	45.4	44.6	46.4	45.2	45.7
Total Lys %	1.10	1.17	1.21	1.09	1.21	1.16	1.09	1.13	1.28	1.18	1.24	1.31
Dig Lys %	1.08	1.11	1.14	1.08	1.11	1.14	1.08	1.11	1.14	1.08	1.11	1.14

1 CAA: control AA density, MAA: medium AA density (+3.0%), HAA: high AA density (+6.0%).

2 Vitamin concentrate supplied per kilogram of diet: retinol, 12000 IU; cholecalciferol, 5000 IU; tocopheryl acetate, 75 mg, menadione, 3 mg; thiamine, 3 mg; riboflavin, 8 mg; niacin, 55 mg; pantothenate, 13 mg; pyridoxine, 5 mg; folate, 2 mg; cyanocobalamin, 16 μg; biotin, 200 μg; cereal-based carrier, 149 mg; mineral oil, 2.5 mg. Trace mineral concentrate supplied per kilogram of diet: Cu (sulphate), 16 mg; Fe (sulphate), 40 mg; I (iodide), 1.25 mg; Se (selenate), 0.3 mg; Mn (sulphate and oxide), 120 mg; Zn (sulphate and oxide), 100 mg; cereal-based carrier, 128 mg; mineral oil, 3.75 mg.

3 Crude protein, crude fat, starch, and total lysine are determined values.

**Table 4:** Ingredients composition and key nutrient profiles of the withdrawal diets (35–42 d).

Ingredients (%)	Control ME (3,150)			ME – 50 kcal (3,100)			ME – 100 kcal (3,050)			ME – 150 Kcal (3,000)		
	CAA^1^	MAA	HAA	CAA	MAA	HAA	CAA	MAA	HAA	CAA	MAA	HAA
Wheat 11.5%	67.49	65.63	63.80	68.68	66.92	65.04	69.93	68.17	66.34	71.66	69.61	67.57
Soybean Meal 46.2%	16.40	18.05	19.70	16.15	17.75	19.40	15.90	17.45	19.10	16.30	17.65	18.95
Canola Seeds	6.00	6.00	6.00	6.00	6.00	6.00	6.00	6.00	6.00	4.25	5.00	5.75
Canola Meal 37.5%	5.00	5.00	5.00	5.00	5.00	5.00	5.00	5.00	5.00	5.00	5.00	5.00
Canola Oil	2.35	2.60	2.80	1.40	1.60	1.85	0.40	0.65	0.85	-	-	-
Limestone	0.82	0.83	0.82	0.83	0.83	0.82	0.83	0.83	0.82	0.83	0.84	0.82
Meat Meal 47 %	0.55	0.50	0.50	0.55	0.50	0.50	0.55	0.50	0.50	0.55	0.50	0.50
Lysine-HCl	0.300	0.295	0.285	0.305	0.300	0.290	0.305	0.305	0.295	0.310	0.305	0.300
Sodium Bicarbonate	0.295	0.290	0.285	0.295	0.295	0.290	0.300	0.295	0.290	0.305	0.300	0.295
DL-Methionine	0.210	0.225	0.240	0.210	0.225	0.235	0.205	0.220	0.235	0.210	0.220	0.235
Vit/Min Premix^2^	0.200	0.200	0.200	0.200	0.200	0.200	0.200	0.200	0.200	0.200	0.200	0.200
Salt	0.155	0.160	0.160	0.155	0.155	0.160	0.150	0.155	0.155	0.150	0.155	0.155
L-Threonine	0.090	0.090	0.090	0.090	0.090	0.090	0.090	0.090	0.090	0.090	0.090	0.090
Choline Chloride	0.050	0.050	0.050	0.050	0.050	0.050	0.050	0.050	0.050	0.050	0.050	0.050
L-Arginine	0.047	0.040	0.032	0.048	0.042	0.034	0.050	0.045	0.037	0.051	0.045	0.039
Xylanase WX 2000	0.010	0.010	0.010	0.010	0.010	0.010	0.010	0.010	0.010	0.010	0.010	0.010
HiPhorius 10G 200 g	0.020	0.020	0.020	0.020	0.020	0.020	0.020	0.020	0.020	0.020	0.020	0.020
Price AUD/T	$635.1	$648.0	$660.9	$614.1	$626.8	$639.8	$593.1	$605.6	$618.6	$576.2	$587.2	$598.3
Nutrients^3^												
AME Kcal/kg	3,150	3,150	3,150	3,100	3,100	3,100	3,050	3050	3050	3000	3000	3000
Crude protein %	19.4	19.9	21.4	20.3	20.6	20.8	19.5	19.9	20.7	19.6	20.7	21.1
Crude fat %	6.21	6.36	6.60	5.10	5.32	5.62	4.15	4.51	4.57	3.15	3.54	3.59
Strach %	46.4	45.7	44.7	46.5	44.7	44.7	45.3	46.6	45.5	47.2	46.8	46.5
Total Lys %	1.03	1.05	1.13	1.06	1.07	1.14	1.03	1.08	1.11	1.06	1.06	1.09
Dig Lys %	1.02	1.05	1.08	1.02	1.05	1.08	1.02	1.05	1.08	1.02	1.05	1.08

1 CAA: control AA density, MAA: medium AA density (+3.0%), HAA: high AA density (+6.0%).

2 Vitamin concentrate supplied per kilogram of diet: retinol, 12000 IU; cholecalciferol, 5000 IU; tocopheryl acetate, 75 mg, menadione, 3 mg; thiamine, 3 mg; riboflavin, 8 mg; niacin, 55 mg; pantothenate, 13 mg; pyridoxine, 5 mg; folate, 2 mg; cyanocobalamin, 16 μg; biotin, 200 μg; cereal-based carrier, 149 mg; mineral oil, 2.5 mg. Trace mineral concentrate supplied per kilogram of diet: Cu (sulphate), 16 mg; Fe (sulphate), 40 mg; I (iodide), 1.25 mg; Se (selenate), 0.3 mg; Mn (sulphate and oxide), 120 mg; Zn (sulphate and oxide), 100 mg; cereal-based carrier, 128 mg; mineral oil, 3.75 mg.

3 Crude protein, crude fat, starch, and total lysine are determined values.

**Table 5:** Broilers growth performance from d 0 to 10 post hatch.

Treatments^1^		BW g/bird		BWG g/bird	FI g/bird	FCR g/g	Feed cost
ME	AA	Day 0	Day 10	0–10 d	0–10 d	0–10 d	$/kg BW
Control	CAA	36.1	318.4	282.3	292.9	1.038^ab^	0.719^a^
Control	MAA	36.6	331.7	295.1	296.3	1.004^d^	0.711^ab^
Control	HAA	36.5	332.9	296.4	286.6	0.967^e^	0.702abc
−50 Kcal	CAA	36.3	326.5	290.1	293.7	1.012bcd	0.680defg
−50 Kcal	MAA	35.9	329.8	293.9	295.8	1.007^cd^	0.693cde
−50 Kcal	HAA	36.1	335.1	299.0	295.6	0.989^de^	0.696bcd
−100 Kcal	CAA	36.3	332.2	295.9	305.3	1.032abc	0.672^fg^
−100 Kcal	MAA	36.1	334.5	298.4	302.5	1.014bcd	0.676efg
−100 Kcal	HAA	35.9	332.0	296.2	298.6	1.008^cd^	0.689cdef
−150 kcal	CAA	36.0	335.1	299.2	313.8	1.049^a^	0.669^g^
−150 kcal	MAA	36.2	337.1	300.9	310.3	1.031abc	0.672^fg^
−150 kcal	HAA	36.3	337.9	301.6	305.5	1.013bcd	0.676efg
	SEM	0.252	2.83	2.73	2.95	0.005	0.003
Main effects							
ME							
Control		36.4	327.7^b^	291.3^b^	291.9^c^	1.003	0.711
−50 Kcal		36.1	330.5^b^	294.3^b^	295.1^c^	1.003	0.689
−100 Kcal		36.1	332.9^ab^	296.8^ab^	302.1^b^	1.018	0.679
−150 Kcal		36.2	336.7^a^	300.6^a^	309.9^a^	1.031	0.672
AA							
CAA		36.2	328.1^b^	291.9^b^	301.4^a^	1.033	0.685
MAA		36.2	333.3^a^	297.1^a^	301.2^a^	1.014	0.688
HAA		36.2	334.5^a^	298.3^a^	296.6^b^	0.994	0.691
Energy		0.465	0.001	0.001	<0.001	<0.001	<0.001
Amino acid		0.983	0.004	0.003	0.036	<0.001	0.127
Energy x Amino acid		0.451	0.129	0.131	0.484	0.001	0.003

Each value for each treatment represents the mean of 8 replicates of 25 birds each.

a-g Means within a column not sharing a superscript differ significantly at the *P* < 0.05 level for the treatment effects and at the P level shown for the main effects.

1 CAA: control amino acid density, MAA: medium amino acid density (+3.0%), HAA: high amino acid density (+6.0%).

### Statistical Analysis

Data were checked for normality and then subjected to statistical analysis using 2-way ANOVA of GLM procedure in JMP®13 (SAS Institute Inc., JMP Software, Cary, NC) to assess the main effects of ME level, AA density, and their interaction. Each pen was considered as an experimental unit and the values presented in the tables are means with pooled standard error of mean (SEM). If a significant effect of treatment was detected, differences between treatments or main effects were separated by Tukey's HSD test. Significance was considered at *P* < 0.05 and *P* < 0.1 was indicated and discussed as a trend.

Based on calculated nutrients, digestible Lys to ME ratios (mg Lys/1000 kcal of ME – DLys:ME) for each diet were determined. Quadratic broken line regression models were fitted to estimate the ideal DLys:ME ratio for optimal BWG and FCR for each period.

## RESULTS

Over the starter period (Table 5), there was no interaction of ME x AA density on BW and FI. Decreasing ME by 150 kcal/kg improved BW, and also independent of ME levels AA at medium and high levels increased BW (*P* < 0.01). Decreasing dietary ME by 100 and 150 kcal increased FI (*P* < 0.01). High AA density decreased FI compared to control and medium levels (*P* < 0.05). Diet energy density at control AA density did not affect FCR, however, an interaction between ME and AA density resulted in improved FCR at medium and high AA densities at control ME level but at −100 and −150 kcal FCR improvement was only significant at high AA density, and no significant improvement of FCR in response to AA density was observed at -50 kcal ME level (*P* < 0.01). The lowest feed cost $/kg BW was calculated for birds receiving diets with -150 Kcal energy at control AA density, which statistically was similar to −50 and −100 kcal at control AA density but significantly lower than control ME at control AA density, resulting in an interaction between ME and AA density (*P* < 0.01).

**Figure 1:**
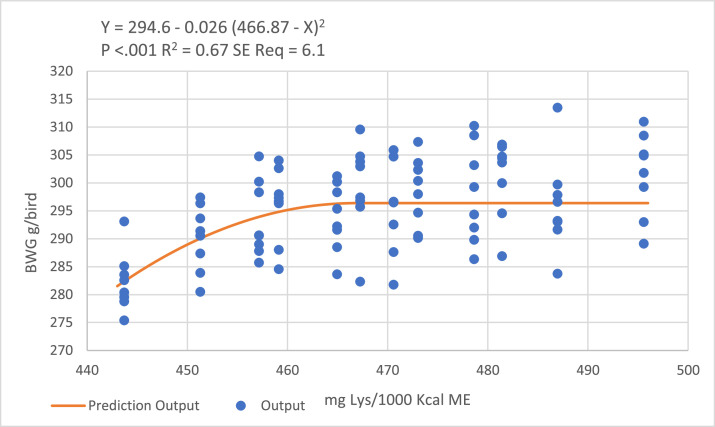
Quadratic broken-line models fitted to describe the relationship between digestible lysine to ME ratio and birds BWG during the starter period.

**Figure 2:**
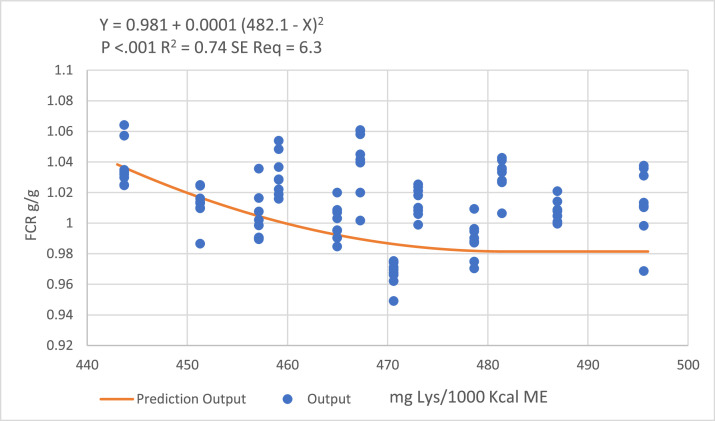
Quadratic broken-line models fitted to describe the relationship between digestible lysine to ME ratio and birds FCR during the starter period.

Based on Figures 1 and 2, fitting quadratic-broken line models predicted 466 and 482 mg DLys/1000 kcal ME to optimize BWG and FCR, respectively, during the starter phase.

Dietary ME and AA levels interacted for BW on d 24 and BWG over the grower period (0–24 d; Table 6). Reducing ME at each level of reduction and at control AA density increased BWG but increasing AA density statistically increased BWG when birds were fed diets with control ME level, and only at high level (+6.0%) increased BWG of the birds’ offered diets with lower ME (*P* < 0.01). Irrespective of AA density, decreasing dietary ME at each level of reduction increased FI which led to a higher FCR by approximately 2.0 points but decreased feed cost $/kg of BW (*P* < 0.01). Increasing AA density, irrespective of ME levels, improved FCR but increased feed cost $/kg of BW (*P* < 0.01).

**Table 6:** Broilers growth performance from d 0 to 24 post hatch.

Treatments		BW g/bird	BWG g/bird	FI g/bird	FCR g/g	Feed cost
ME	AA^1^	Day 24	0–24 d	0–24 d	0–24 d	$/kg BW
Control	CAA	1,395^f^	1359^f^	1,688	1.242	0.838
Control	MAA	1,469abcd	1433abcd	1,753	1.224	0.847
Control	HAA	1,476abc	1439abc	1,731	1.203	0.847
−50 Kcal	CAA	1,437^de^	1400^de^	1,751	1.250	0.818
−50 Kcal	MAA	1,442cde	1406cde	1,746	1.242	0.832
−50 Kcal	HAA	1,482^ab^	1446^ab^	1,760	1.217	0.831
−100 Kcal	CAA	1,432^e^	1396^e^	1,788	1.281	0.812
−100 Kcal	MAA	1,461abcde	1424abcde	1,791	1.257	0.826
−100 Kcal	HAA	1,488^a^	1452^a^	1,800	1.239	0.821
−150 kcal	CAA	1,433^e^	1397^e^	1,803	1.290	0.798
−150 kcal	MAA	1,449bcde	1413bcde	1,802	1.276	0.805
−150 kcal	HAA	1,479^ab^	1443^ab^	1,819	1.261	0.810
	SEM	7.22	7.15	14.08	0.007	0.005
Main effects						
ME						
Control		1,447	1,410	1,723^b^	1.222^c^	0.844^a^
−50 Kcal		1,454	1,417	1,752^b^	1.236^c^	0.827^b^
−100 Kcal		1,460	1,424	1,792^a^	1.259^b^	0.819^b^
−150 Kcal		1,454	1,417	1,809^a^	1.275^a^	0.804^c^
AA						
CAA		1,424	1,388	1,757	1.265^a^	0.817^b^
MAA		1,455	1,419	1,773	1.249^b^	0.827^a^
HAA		1,481	1,445	1,777	1.229^c^	0.827^a^
Energy		0.161	0.146	<0.001	<0.001	<.001
Amino acid		<0.001	<0.001	0.110	<0.001	0.002
Energy x Amino acid		0.006	0.006	0.212	0.140	0.957

Each value for each treatment represents the mean of 8 replicates of 25 birds each.

a-f Means within a column not sharing a superscript differ significantly at the *P* < 0.05 level for the treatment effects and at the P level shown for the main effects.

1 CAA: control amino acid density, MAA: medium amino acid density (+3.0%), HAA: high amino acid density (+6.0%).

**Figure 3:**
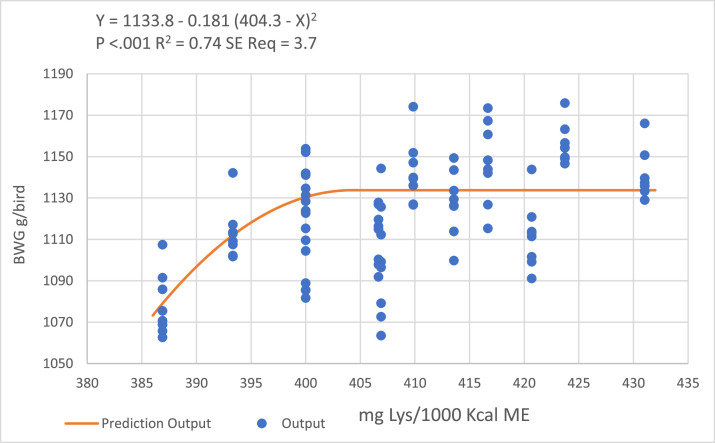
Quadratic broken-line models fitted to describe the relationship between digestible lysine to ME ratio and birds BWG during the grower period.

**Figure 4:**
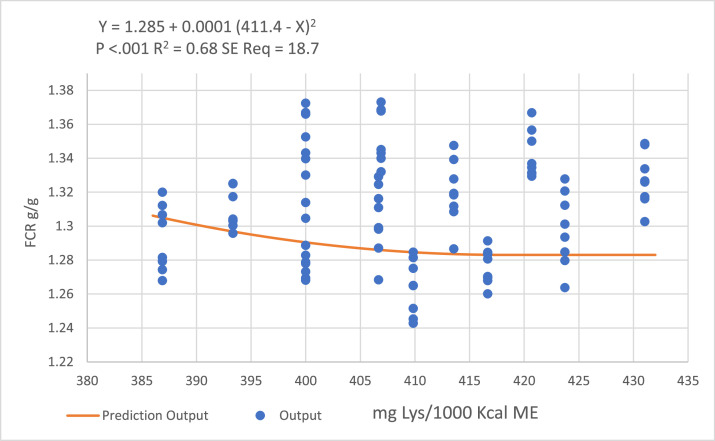
Quadratic broken-line models fitted to describe the relationship between digestible lysine to ME ratio and birds FCR during the grower period.

Based on the Figures 3 and 4, quadratic-broken line regression models predicted 404 and 411 mg DLys/1000 kcal ME to optimize BWG and FCR, respectively, during the grower period.

As shown in Table 7, an interaction between dietary ME and AA levels led to a greater BW on d 35 and higher BWG (0–35 d) in response to medium and high AA compared to control AA in birds fed diets with control ME compared to reduced ME diets (*P* < 0.01). Reducing dietary ME (*P* < 0.01) or increasing AA density (*P* < 0.05), independently, increased FI during the finisher period. However, the higher FI in low ME diets resulted in a higher FCR at each level of ME reduction, while the higher AA density at both levels decreased FCR (*P* < 0.01). Decreasing ME density reduced feed cost $/kg BW and increasing AA density, particularly at +6%, increased the cost (*P* < 0.01).

**Table 7:** Broilers growth performance from d 0 to 35 post hatch.

Treatments		BW g/bird	BWG g/bird	FI g/bird	FCR g/g	Feed cost
ME	AA^1^	Day 35	0–35 d	0–35 d	0–35 d	S/kg BW
Control	CAA	2690^f^	2654^f^	3,680	1.387	0.912
Control	MAA	2816abc	2779abc	3,751	1.350	0.908
Control	HAA	2833^ab^	2797^ab^	3,712	1.327	0.910
−50 Kcal	CAA	2743def	2707def	3,752	1.386	0.884
−50 Kcal	MAA	2763cde	2727cde	3,773	1.383	0.901
−50 Kcal	HAA	2851^a^	2815^a^	3,835	1.362	0.905
−100 Kcal	CAA	2740def	2704def	3,812	1.410	0.869
−100 Kcal	MAA	2791bcd	2755bcd	3,823	1.388	0.880
−100 Kcal	HAA	2832^ab^	2796^ab^	3,844	1.375	0.884
−150 kcal	CAA	2725^ef^	2689^ef^	3,840	1.428	0.859
−150 kcal	MAA	2793abcd	2757abcd	3,879	1.407	0.862
−150 kcal	HAA	2814abc	2777abc	3,875	1.395	0.871
	SEM	12.43	12.32	26.31	0.007	0.004
Main effects						
ME						
Control		2,780	2,743	3,714^c^	1.354^c^	0.910^a^
−50 Kcal		2,786	2,749	3,786^b^	1.377^b^	0.896^b^
−100 Kcal		2,788	2,751	3,826^ab^	1.391^b^	0.877^c^
−150 Kcal		2,777	2,741	3,864^a^	1.410^a^	0.863^d^
AA						
CAA		2,725	2,688	3,771^b^	1.402^a^	0.881^b^
MAA		2,791	2,754	3,806^ab^	1.382^b^	0.887^ab^
HAA		2,833	2,796	3,816^a^	1.365^c^	0.892^a^
*P-value*						
Energy		0.691	0.674	<0.001	<0.001	<0.001
Amino acid		<0.001	<0.001	0.041	<0.001	0.002
Energy x Amino acid		0.001	0.001	0.617	<0.167	0.182

Each value for each treatment represents the mean of 8 replicates of 25 birds each.

a-f Means within a column not sharing a superscript differ significantly at the *P* < 0.05 level for the treatment effects and at the P level shown for the main effects.

1 CAA: control amino acid density, MAA: medium amino acid density (+3.0%), HAA: high amino acid density (+6.0%).

**Figure 5:**
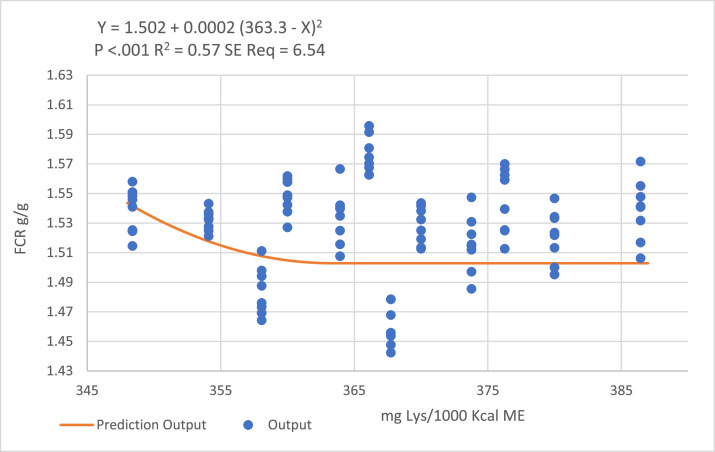
Quadratic broken-line models fitted to describe the relationship between digestible lysine to ME ratio and birds FCR during the finisher period.

Quadratic-broken line regression models could not be fitted to predict a DLys:ME ratio to optimize BWG, but the models predicted a ratio of 363 mg DLys/1000 kcal ME to optimize FCR for the finisher period (24–35 d; Figure 5).

Table 8 summarizes the effect of dietary treatment on birds’ productive traits over the entire production period (0-42 d). ME reduction at control AA density had no significant effect on final BW (d 42) and BWG (0–42 d). However, dietary ME and AA levels interacted for the final BW and BWG, where both medium and high AA density significantly increased BWG compared to control AA density when birds were fed diets with control, −100 and −150 kcal ME, while AA density at medium levels with −50 kcal ME did not statistically affect BW or BWG at d 42 (*P* < 0.05). Reducing ME density, regardless of AA density, increased the overall FI (*P* < 0.01). Similarly, increasing AA density, independent of ME levels, increased FI (*P* < 0.05). An interaction between ME and AA density resulted in improved FCR, and bodyweight corrected FCR, in response to increased AA density at both medium and high levels only at control ME density, but with reduced ME diets FCR significantly improved only when birds received high AA density diets (*P* < 0.01). The ME levels did not affect average age to 2.5 kg of BW (*P* > 0.05). Increasing AA density at both medium and high levels decreased average age to 2.5 kg of BW at each level of ME reduction, except at −50 Kcal ME and at medium AA density, which resulted in an interaction between ME and AA density (*P* < 0.01). Decreasing ME density, at each level of reduction, decreased feed cost $/kg of BW, while increasing the AA density increased the cost (*P* < 0.01).

**Table 8:** Broilers growth performance over the entire production period from d 0 to 42 post hatch.

Treatments		BW g/bird	BWG g/bird	FI g/bird	FCR g/g	BWc FCR	Age to 2.5 kg	Feed cost
ME	AA^1^	D 42	0-42 d	0-42 d	0-42 d	0-42 d	D	$/kg BW
Control	CAA	3,492^f^	3,455^f^	5,045	1.460^e^	1.460^de^	30.4^a^	0.951
Control	MAA	3,647^ab^	3,610^ab^	5,207	1.442^f^	1.411^g^	29.1def	0.960
Control	HAA	3,685^a^	3,649^a^	5,200	1.425^g^	1.386^h^	28.8^f^	0.967
−50 Kcal	CAA	3,534def	3,498def	5,187	1.483^cd^	1.474^cd^	30.0abc	0.936
−50 Kcal	MAA	3,571cde	3,535cde	5,248	1.485^cd^	1.469^cd^	29.7bcd	0.956
−50 Kcal	HAA	3,671^ab^	3,635^ab^	5,317	1.463^e^	1.427^fg^	28.9^f^	0.961
−100 Kcal	CAA	3,529^ef^	3,492^ef^	5,265	1.507^ab^	1.500^b^	30.1^ab^	0.920
−100 Kcal	MAA	3,602bcd	3,566bcd	5,303	1.487^cd^	1.465^d^	29.4cde	0.931
−100 Kcal	HAA	3,647^ab^	3,612^ab^	5,326	1.475^de^	1.443^ef^	29.1^ef^	0.938
−150 kcal	CAA	3,509^ef^	3473^ef^	5,293	1.524^a^	1.520^a^	30.2^ab^	0.906
−150 kcal	MAA	3,601bcd	3,565bcd	5,374	1.508^ab^	1.486^bc^	29.5cde	0.913
−150 kcal	HAA	3,640abc	3,604abc	5,399	1.498^bc^	1.468^cd^	29.1^ef^	0.924
	SEM	14.89	14.79	27.19	0.004	0.004	0.123	0.002
Main effects								
ME								
Control		3,608	3,571	5,151^c^	1.442	1.419	29.4	0.960^a^
−50 Kcal		3,592	3,556	5,251^b^	1.477	1.457	29.5	0.951^b^
−100 Kcal		3,593	3,557	5,298^ab^	1.490	1.469	29.5	0.930^c^
−150 Kcal		3,583	3,547	5,356^a^	1.510	1.491	29.6	0.914^d^
AA								
CAA		3,516	3,480	5,198^b^	1.494	1.489	30.2	0.928^c^
MAA		3,605	3,569	5,283^a^	1.480	1.458	29.4	0.940^b^
HAA		3661	3625	5311^a^	1.465	1.431	29.0	0.948^a^
Energy		0.251	0.255	<0.001	<0.001	<0.001	0.296	<0.001
Amino acid		<0.001	<0.001	<0.001	<0.001	<0.001	<0.001	<0.001
Energy x Amino acid		0.005	0.005	0.292	0.045	<0.001	0.005	0.081

a-f Means within a column not sharing a superscript differ significantly at the *P* < 0.05 level for the treatment effects and at the P level shown for the main effects.

1 CAA: control amino acid density, MAA: medium amino acid density (+3.0%), HAA: high amino acid density (+6.0%).

**Figure 6:**
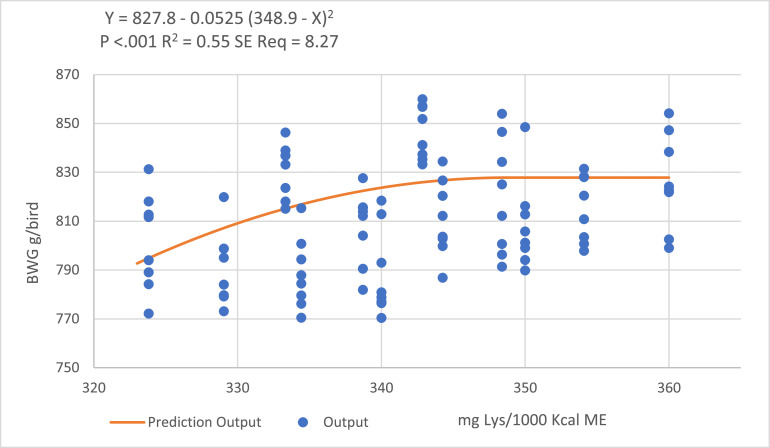
Quadratic broken-line models fitted to describe the relationship between digestible lysine to ME ratio and birds BWG during the withdrawal period.

**Figure 7:**
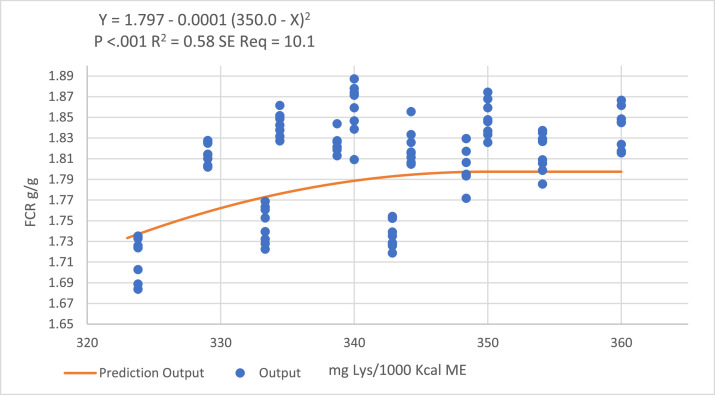
Quadratic broken-line models fitted to describe the relationship between digestible lysine to ME ratio and birds FCR during the withdrawal period.

Quadratic-broken line regression models predicted a ratio of 345 DLys/1,000 kcal ME to optimize BWG, but as increasing the DLys:ME ratio in the withdrawal period increased feed intake and consequently the FCR, the models failed to fit a descending line to predict optimal DLys:ME ratio for optimal FCR (Figures 6 and 7).

## DISCUSSION

### Growth Performance

For many years, NRC (1994) remained the main reference point for poultry nutritionists and researchers to formulate a balanced diet for both broiler chickens and laying hens. However, since mid-2000s the breeder companies began publishing their own recommended nutrient specifications dedicated to certain commercial breeds and strains of birds. These recommendations are revised and updated every couple of years to manifest the latest genetic improvements. In line with published research data (Macelline et al., 2023; Maynard et al., 2019; Sharma et al., 2018) an increase in AA density and a decrease in ME has been a fixed trend in every new set of nutrient recommendations published, at least for broiler chickens. For instance, Aviagen recommendation for digestible Lys in finisher diet has increased from 0.97% in 2007 to 1.08% in 2022; meanwhile, the ME density has decreased from 3,200 to 3,100 kcal/kg within the same period (Aviagen, 2007 and 2022).

In the present study, the response to dietary ME density was age-dependent, where in terms of BWG younger birds (up to 24 d) responded positively to reduced ME density, and at control AA density, gained higher weight compared to their counterparts fed diets with higher ME density. While, increasing AA density improved BWG and FCR irrespective of age. Since the ME required for maintenance represents a significant proportion of the total ME intake, this could explain the lack of response in younger birds to higher dietary ME (control ME). At 35 and 42 d post-hatch, both ME and AA density influenced FI independently. At these ages, even a modest 50 kcal reduction in ME density was sufficient to impact FI. Poultry, like mammals, have complex mechanisms regulating feed intake (Denbow, 1999). In poultry species, BW is maintained throughout the lifecycle by adjustments of FI and energy expenditure. Both processes are controlled by complex and interconnected neuronal and endocrine networks that function to achieve energy homeostasis and maintain BW (Richards and Proszkowiec-Weglarz, 2007). Although compensation may not be complete - depending on the degree of a particular nutrient restriction/deficiency - but birds are able to increase their intake to compensate for the restricted/deficient nutrient i.e. ME or AA, in order to support their BW. However, in this study the higher intake in response to reduced ME could be a function of both higher BW and also the lower ME density of the diet at a certain age. All measurements of ME and AA balance are positive curvilinear functions of body mass. Total metabolism of the bird increases exponentially with body mass; hence ME and AA requirements increase to support both maintenance and growth. Interestingly, the interactions between ME and AA density for BW were more pronounced in older birds of a higher body mass. Such interactions resulted in distinct responses to AA density within control (higher) ME density, rather at lower ME densities. This confirms the hypothesis that at high AA intake, a higher supply/intake of ME is required for the birds to utilize the increased AA ingestion and convert it into muscle mass. The partitioning of ME and AA for maintenance and muscle growth will certainly be affected by their balance (Classen, 2017) and birds’ age (Aftab, 2019). The experimental diets, regardless of ME or AA densities, were balanced based on ideal digestible AA ratios. Thus, at high AA intake and supply of adequate ME (control ME diets), especially in older birds with a bigger body mass, less AA could have been catabolized to meet the ME requirements and more were directed towards growth and muscle tissue accretion, resulting in higher feed efficiency.

Looking at growth performance over the entire production period (0–42 d) similar interactive trends between ME and AA densities were observed, except for FI, which independently increased in response to reduced ME or increased AA density. Overall, accumulative reduction in ME did not affect BWG but each 50-kcal reduction in ME increased FCR by around 2.0 points. Similar to our results, a recent study by (Maharjan et al., 2021a) showed significant reduction in feed intake (61 g/bird) and improved FCR by 7.3 points when dietary ME was increased by 125 kcal/kg. In our study, increasing AA density accelerated growth rate by nearly half a day and improved FCR by 3 points for each 3.0% increase in density. Despite the different responses to reduction of dietary ME and increased AA density, the overall feed cost per kg of BW linearly decreased as dietary ME decreased but the cost increased as AA density increased. Indicating that the improved growth rate and feed efficiency with higher AA density *per se* did not cover the initial cost investment in dietary ingredients.

### Digestible Lys to ME Ratio

In practical feed formulation, the optimal utilization of dietary ME and AA by birds is a high priority issue for environmental and economic concerns. Therefore, changes in ME and AA concentrations should be considered proportionally together or a limit in lean tissue deposition may be reached to store or disperse energy when excess energy is fed (Barekatain et al., 2021). In addition, when amino acids are independently increased, they may increasingly be used as energy sources (Gous et al., 2018; Richards and Proszkowiec-Weglarz, 2007). Hence, digestible lysine to ME ratio (**DLys:ME**) has long been introduced to ensure that changes in ME or AA densities are proportional. In current study, quadratic broken line modes (**QBL**) were fitted to predict DLys:ME ratios required to optimize BWG and FCR. In starter and grower phases, QBL models estimated a higher DLys:ME ratio for optimal FCR than BWG, and both estimates were higher than the breeder recommendations (Aviagen, 2022). Although QBL could not be fitted to predict the DLys:ME ratio required to optimize BWG in finisher and FCR in withdrawal phases, yet the estimated DLys:ME for optimal FCR and BWG in finisher and withdrawal phases, respectively, were higher than the breeder recommendations (Aviagen, 2022). The positive effect of increasing DLys:ME ratio on performance parameters in broiler chickens is well documented (Barekatain et al., 2021; Mansilla et al., 2022). Maharjan et al. (2021b) estimated both the essential and nonessential digestible AA requirements for optimal performance in relation to BWG, FCR, and breast meat yield for broilers grown at thermoneutral environment. Similar to our findings, the authors reported higher DLys:ME ratios compared to the existing breeder recommendations. Interestingly and in line with the findings of the current study, the requirements of all AA (essential and nonessential) to optimize FCR was higher than the levels needed to optimize BWG. This also agrees with the work by (Liu et al., 2019) reporting a dietary digestible Lys requirement of 1.03% for optimal BWG and 1.22% for FCR during the early finishing feeding phase of male broilers.

## CONCLUSIONS

In summary, the findings of the current study indicate that decreasing dietary ME does not have any significant effect on BWG and growth rate. In fact, younger birds, with a smaller body mass and hence lower energy requirements for maintenance, could benefit from low ME diets. However, accumulatively every 50 kcal/kg reduction in ME increases feed intake by around 66 g/bird (0–42 d). This increased FI in response to lower ME increases FCR by around 2.0 (0.02) points per every 50-kcal reduction of ME across all AA levels. Each 3.0 % increase in AA density improves FCR by around 3.0 (0.03) points. Under commercial conditions, depending on the availability and prices of energy and protein sources and when feed intake is not a limiting factor, reducing dietary ME concentration could be used as a nutritional tool to optimize feed cost $/kg of bodyweight.

Quadratic broken line models suggest optimal digestible Lys to ME ratios of 474 and 407 mg DLys/1000 kcal ME for starter and grower diets, respectively, while the finisher diets should have a ratio of 363 for optimal FCR and the withdrawal diets a ratio of 349 for optimal BWG. These ratios optimize performance but may not minimize costs, so decisions regarding the dietary nutrient density should consider input-output relationships and costs.

## DISCLOSURES

We declare that we have no financial and personal relationships with other people or organizations that can inappropriately influence our work, and there is no professional or other personal interest of any nature or kind in any product, service and/or company that could be construed as influencing the content of this paper.

## Appendix Supplementary materials

Image, application 1
