# Supplementary material for: Interactive effects of dietary energy levels with amino acid density on growth performance and optimal digestible Lys to energy ratio of male broiler chickens
Source: Poult Sci. 2024 Sep 26;103(12):104361. doi: 10.1016/j.psj.2024.104361 (PMC11525130; doi:10.1016/j.psj.2024.104361)
Supplement: Supplementary file 1 [file mmc1.docx]

**Table S1.** Calculated and measured nutrient content of the starter diets (0-10 d). Values in parentheses are analyzed values and for amino acids represents total not digestible.

| Nutrients | Control ME (2975) | | | ME – 50 kcal (2925) | | | ME – 100 kcal (2875) | | | ME – 150 Kcal (2825) | | |
| --- | --- | --- | --- | --- | --- | --- | --- | --- | --- | --- | --- | --- |
|  | CAA^1^ | MAA | HAA | CAA | MAA | HAA | CAA | MAA | HAA | CAA | MAA | HAA |
| AME Kcal/kg | 2975 | 2975 | 2975 | 2925 | 2925 | 2925 | 2875 | 2875 | 2875 | 2825 | 2825 | 2825 |
| GE Kcal/kg | 3993 | 3985 | 4020 | 3923 | 3933 | 3954 | 3862 | 3876 | 3867 | 3807 | 3821 | 3825 |
| DLys/ME ratio^2^ | 444 | 457 | 471 | 451 | 465 | 479 | 459 | 473 | 487 | 467 | 481 | 496 |
| Moisture % | 9.73 | 9.66 | 9.59 | 9.84 | 9.77 | 9.70 | 9.93 | 9.88 | 9.82 | 9.92 | 9.89 | 9.85 |
| Crude protein % | 24.4 (24.1) | 25.0 (25.2) | 25.6 (25.3) | 24.5 (24.1) | 25.0 (24.8) | 25.6 (24.9) | 24.5 (24.6) | 25.1 (24.7) | 25.6 (25.2) | 24.6 (24.5) | 25.1 (25.2) | 25.7 (25.3) |
| Dig.Lys^3^ % | 1.32 (1.34) | 1.36 (1.41) | 1.40 (1.44) | 1.32 (1.37) | 1.36 (1.39) | 1.40 (1.44) | 1.32 (1.40) | 1.36 (1.44) | 1.40 (1.45) | 1.32 (1.38) | 1.36 (1.41) | 1.40 (1.45) |
| Dig.Met + Cys % | 1.00 | 1.03 | 1.07 | 1.00 | 1.03 | 1.07 | 1.00 | 1.03 | 1.06 | 1.00 | 1.03 | 1.07 |
| Dig.Thr % | 0.89 (0.91) | 0.91 (0.97) | 0.94 (1.00) | 0.88 (0.90) | 0.91 (0.96) | 0.94 (1.00) | 0.88 (0.95) | 0.91 (0.97) | 0.94 (0.99) | 0.88 (0.94) | 0.91 (0.97) | 0.94 (1.01) |
| Dig.Ile % | 0.90 (0.93) | 0.93 (0.98) | 0.95 (1.01) | 0.90 (0.91) | 0.93 (0.98) | 0.95 (1.02) | 0.90 (0.97) | 0.93 (0.99) | 0.95 (1.00) | 0.90 (0.97) | 0.93 (0.98) | 0.95 (0.99) |
| Dig.Leu % | 1.54 (1.60) | 1.58 (1.69) | 1.62 (1.73) | 1.54 (1.58) | 1.58 (1.69) | 1.62 (1.71) | 1.54 (1.62) | 1.58 (1.73) | 1.62 (1.75) | 1.55 (1.68) | 1.59 (1.69) | 1.63 (1.69) |
| Dig.Trp % | 0.26 | 0.27 | 0.28 | 0.26 | 0.27 | 0.28 | 0.26 | 0.27 | 0.28 | 0.26 | 0.27 | 0.28 |
| Dig.Arg % | 1.41 (1.48) | 1.46 (1.49) | 1.50 (1.54) | 1.41 (1.44) | 1.46 (1.47) | 1.50 (1.52) | 1.41 (1.42) | 1.46 (1.48) | 1.50 (1.55) | 1.41 (1.42) | 1.46 (1.50) | 1.50 (1.56) |
| Dig.His % | 0.53 (0.56) | 0.54 (0.59) | 0.55 (0.61) | 0.53 (0.57) | 0.54 (0.59) | 0.55 (0.60) | 0.53 (0.59) | 0.54 (0.60) | 0.55 (0.60) | 0.53 (0.59) | 0.54 (0.59) | 0.55 (0.61) |
| Dig.Val % | 1.00 (1.07) | 1.03 (1.11) | 1.07 (1.15) | 1.00 (1.04) | 1.03 (1.12) | 1.07 (1.16) | 1.00 (1.05) | 1.03 (1.12) | 1.06 (1.15) | 1.00 (1.11) | 1.03 (1.12) | 1.07 (1.16) |
| Crude Fat % | 4.65 (4.71) | 4.89 (5.02) | 5.14 (5.22) | 3.70 (4.01) | 3.95 (4.22) | 4.20 (4.28) | 2.70 (3.02) | 3.00 (3.25) | 3.25 (3.42) | 1.80 (2.11) | 2.07 (2.31) | 2.31 (2.39) |
| Crude Fiber % | 3.21 | 3.23 | 3.25 | 3.23 | 3.25 | 3.27 | 3.16 | 3.27 | 3.29 | 3.01 | 3.10 | 3.18 |
| Starch % | 40.3 (41.1) | 39.2 (40.5) | 38.0 (38.8) | 41.0 (41.7) | 39.9 (41.0) | 38.8 (39.6) | 41.9 (42.1) | 40.6 (41.3) | 39.5 (40.4) | 42.5 (43.1) | 41.3 (41.8) | 40.2 (41.1) |
| Phytate P % | 0.25 | 0.25 | 0.25 | 0.25 | 0.25 | 0.25 | 0.25 | 0.25 | 0.25 | 0.25 | 0.25 | 0.25 |
| Ca % | 0.95 (0.84) | 0.95 (0.87) | 0.95 (0.82) | 0.95 (0.79) | 0.95 (0.84) | 0.95 (0.81) | 0.95 (0.85) | 0.95 (0.80) | 0.95 (0.84) | 0.95 (0.83) | 0.95 (0.84) | 0.95 (0.85) |
| Available P % | 0.50 | 0.50 | 0.50 | 0.50 | 0.50 | 0.50 | 0.50 | 0.50 | 0.50 | 0.50 | 0.50 | 0.50 |
| Total P % | 0.60 (0.58) | 0.60 (0.62) | 0.60 (0.63) | 0.60 (0.65) | 0.60 (0.62) | 0.60 (0.64) | 0.60 (0.59) | 0.60 (0.61) | 0.60 (0.63) | 0.60 (0.65) | 0.60 (0.58) | 0.60 (0.63) |
| Na % | 0.20 (0.16) | 0.20 (0.17) | 0.20 (0.18) | 0.20 (0.18) | 0.20 (0.16) | 0.20 (0.16) | 0.20 (0.17) | 0.20 (0.18) | 0.20 (0.16) | 0.20 (0.17) | 0.20 (0.18) | 0.20 (0.19) |
| Cl % | 0.24 | 0.24 | 0.24 | 0.24 | 0.24 | 0.24 | 0.24 | 0.24 | 0.24 | 0.24 | 0.24 | 0.24 |
| K % | 0.84 (0.87) | 0.86 (0.92) | 0.89 (0.93) | 0.84 (0.84) | 0.87 (0.95) | 0.89 (0.94) | 0.84 (0.88) | 0.87 (0.91) | 0.89 (0.96) | 0.84 (0.86) | 0.86 (0.92) | 0.89 (0.92) |
| Na+K-Cl meq/kg | 234 | 240 | 247 | 234 | 241 | 247 | 234 | 241 | 247 | 234 | 240 | 247 |

^1^CAA: control AA density, MAA: medium AA density (+3.0%), HAA: high AA density (+6.0%);

^2^ mg digestible Lys per 1000 kcal of ME

^3^ Digestible

**Table S2.** Calculated and measured nutrient content of the grower diets (10-24 d). Values in parentheses are analyzed values and for amino acids represents total not digestible.

| Nutrients | Control ME (3050) | | | ME – 50 kcal (3000) | | | ME – 100 kcal (2950) | | | ME – 150 Kcal (2900) | | |
| --- | --- | --- | --- | --- | --- | --- | --- | --- | --- | --- | --- | --- |
|  | CAA^1^ | MAA | HAA | CAA | MAA | HAA | CAA | MAA | HAA | CAA | MAA | HAA |
| AME Kcal/kg | 3050 | 3050 | 3050 | 3000 | 3000 | 3000 | 2950 | 2950 | 2950 | 2900 | 2900 | 2900 |
| GE kcal/kg | 4072 | 4098 | 4092 | 4012 | 4001 | 3998 | 3940 | 3960 | 3965 | 3886 | 3867 | 3911 |
| Lys/ME ratio^2^ | 387 | 400 | 410 | 393 | 407 | 417 | 400 | 413 | 424 | 407 | 421 | 431 |
| Moisture % | 9.74 | 9.67 | 9.62 | 9.85 | 9.78 | 9.73 | 9.96 | 9.89 | 9.84 | 10.02 | 9.98 | 9.95 |
| Crude protein % | 22.4 (22.2) | 23.0 (23.3) | 23.4 (23.6) | 22.5 (22.4) | 23.0 (22.8) | 23.4 (23.7) | 22.5 (22.7) | 23.1 (23.5) | 23.5 (23.4) | 22.5 (22.4) | 23.1 (23.6) | 23.5 (23.7) |
| Dig.Lys^3^ % | 1.18 (1.22) | 1.22 (1.31) | 1.25 (1.35) | 1.18 (1.26) | 1.22 (1.32) | 1.25 (1.33) | 1.18 (1.30) | 1.22 (1.32) | 1.25 (1.38) | 1.18 (1.26) | 1.22 (1.31) | 1.25 (1.36) |
| Dig.Met + Cys % | 0.92 | 0.95 | 0.98 | 0.92 | 0.95 | 0.98 | 0.92 | 0.95 | 0.98 | 0.92 | 0.95 | 0.98 |
| Dig.Thr % | 0.79 (0.85) | 0.82 (0.90) | 0.84 (0.92) | 0.79 (0.85) | 0.82 (0.90) | 0.84 (0.89) | 0.79 (0.83) | 0.82 (0.88) | 0.84 (0.94) | 0.79 (0.84) | 0.82 (0.86) | 0.84 (0.91) |
| Dig.Ile % | 0.81 (0.87) | 0.84 (0.92) | 0.86 (0.95) | 0.81 (0.87) | 0.84 (0.91) | 0.86 (0.91) | 0.81 (0.88) | 0.84 (0.90) | 0.86 (0.96) | 0.82 (0.87) | 0.84 (0.87) | 0.86 (0.94) |
| Dig.Leu % | 1.42 (1.52) | 1.46 (1.59) | 1.49 (1.64) | 1.42 (1.55) | 1.46 (1.58) | 1.49 (1.59) | 1.43 (1.56) | 1.47 (1.57) | 1.50 (1.66) | 1.43 (1.52) | 1.47 (1.52) | 1.50 (1.63) |
| Dig.Trp % | 0.24 | 0.25 | 0.26 | 0.24 | 0.25 | 0.26 | 0.24 | 0.25 | 0.26 | 0.25 | 0.25 | 0.26 |
| Dig.Arg % | 1.28 (1.31) | 1.32 (1.35) | 1.35 (1.39) | 1.27 (1.32) | 1.32 (1.34) | 1.35 (1.39) | 1.27 (1.29) | 1.32 (1.33) | 1.35 (1.38) | 1.27 (1.29) | 1.32 (1.35) | 1.35 (1.36) |
| Dig.His % | 0.49 (0.53) | 0.50 (0.56) | 0.51 (0.57) | 0.49 (0.56) | 0.50 (0.53) | 0.51 (0.55) | 0.49 (0.55) | 0.50 (0.55) | 0.51 (0.58) | 0.49 (0.53) | 0.50 (0.53) | 0.51 (0.57) |
| Dig.Val % | 0.91 (1.01) | 0.94 (1.04) | 0.96 (1.08) | 0.91 (0.99) | 0.94 (1.04) | 0.96 (1.04) | 0.91 (1.03) | 0.94 (1.03) | 0.96 (1.09) | 0.91 (1.00) | 0.94 (1.01) | 0.96 (1.07) |
| Crude Fat % | 5.63 (5.85) | 5.88 (5.89) | 6.06 (5.97) | 4.69 (4.55) | 4.93 (4.95) | 5.12 (5.21) | 3.75 (3.88) | 3.99 (4.15) | 4.18 (4.19) | 2.66 (2.78) | 2.98 (3.12) | 3.23 (3.25) |
| Crude Fiber % | 3.32 | 3.34 | 3.36 | 3.34 | 3.36 | 3.37 | 3.35 | 3.38 | 3.39 | 3.17 | 3.30 | 3.41 |
| Starch % | 41.8 (41.5) | 40.7 (41.1) | 39.8 (40.7) | 42.6 (42.8) | 41.4 (42.6) | 40.6 (41.3) | 43.3 (42.8) | 42.2 (42.5) | 41.3 (40.8) | 44.4 (43.7) | 43.1 (43.7) | 42.1 (42.3) |
| Phytate P % | 0.25 | 0.25 | 0.25 | 0.25 | 0.25 | 0.25 | 0.25 | 0.25 | 0.25 | 0.25 | 0.25 | 0.25 |
| Ca % | 0.85 (0.81) | 0.85 (0.77) | 0.85 (0.74) | 0.85 (0.78) | 0.85 (0.78) | 0.85 (0.77) | 0.85 (0.80) | 0.85 (0.75) | 0.85 (0.74) | 0.85 (0.75) | 0.85 (0.77) | 0.85 (0.76) |
| Avai P % | 0.43 | 0.43 | 0.43 | 0.43 | 0.43 | 0.43 | 0.43 | 0.43 | 0.43 | 0.43 | 0.43 | 0.43 |
| Total P % | 0.50 (0.55) | 0.50 (0.52) | 0.50 (0.54) | 0.50 (0.57) | 0.50 (0.52) | 0.51 (0.57) | 0.50 (0.57) | 0.51 (0.55) | 0.51 (0.56) | 0.50 (0.57) | 0.51 (0.58) | 0.51 (0.56) |
| Na % | 0.20 (0.16) | 0.20 (0.18) | 0.20 (0.17) | 0.20 (0.17) | 0.20 (0.16) | 0.20 (0.18) | 0.20 (0.16) | 0.20 (0.17) | 0.20 (0.16) | 0.20 (0.18) | 0.20 (0.16) | 0.20 (0.18) |
| Cl % | 0.23 | 0.23 | 0.23 | 0.23 | 0.23 | 0.23 | 0.23 | 0.23 | 0.23 | 0.23 | 0.23 | 0.23 |
| K % | 0.78 (0.84) | 0.81 (0.87) | 0.82 (0.85) | 0.78 (0.80) | 0.81 (0.85) | 0.82 (0.88) | 0.78 (0.86) | 0.81 (0.85) | 0.83 (0.87) | 0.78 (0.84) | 0.81 (0.86) | 0.83 (0.88) |
| Na+K-Cl meq/kg | 222 | 228 | 233 | 222 | 228 | 233 | 222 | 228 | 233 | 222 | 228 | 233 |

^1^CAA: control AA density, MAA: medium AA density (+3.0%), HAA: high AA density (+6.0%);

^2^ mg digestible Lys per 1000 kcal of ME

^3^ Digestible

**Table S3.** Calculated and measured nutrient content of the finisher diets (24-35 d). Values in parentheses are analyzed values and for amino acids represents total not digestible.

| Nutrients | Control ME (3100) | | | ME – 50 kcal (3050) | | | ME – 100 kcal (3000) | | | ME – 150 Kcal (2950) | | |
| --- | --- | --- | --- | --- | --- | --- | --- | --- | --- | --- | --- | --- |
|  | CAA^1^ | MAA | HAA | CAA | MAA | HAA | CAA | MAA | HAA | CAA | MAA | HAA |
| AME Kcal/kg | 3100 | 3100 | 3100 | 3050 | 3050 | 3050 | 3000 | 3000 | 3000 | 2950 | 2950 | 2950 |
| GE kcal/kg | 4078 | 4094 | 4110 | 4021 | 4005 | 4012 | 3963 | 3965 | 3961 | 3868 | 3880 | 3894 |
| Lys/ME ratio^2^ | 348 | 358 | 368 | 354 | 364 | 374 | 360 | 370 | 380 | 366 | 376 | 386 |
| Moisture % | 9.95 | 9.89 | 9.83 | 10.06 | 10.01 | 9.95 | 10.17 | 10.12 | 10.06 | 10.19 | 10.16 | 10.13 |
| Crude protein % | 20.9 (20.2) | 21.4 (20.8) | 21.9 (21.6) | 21.0 (21.3) | 21.4 (21.6) | 21.9 (21.8) | 21.0 (21.3) | 21.4 (21.9) | 21.9 (22.5) | 21.0 (21.4) | 21.5 (21.7) | 22.0 (22.7) |
| Dig.Lys^3^ % | 1.08 (1.10) | 1.11 (1.17) | 1.14 (1.21) | 1.08 (1.09) | 1.11 (1.21) | 1.14 (1.16) | 1.08 (1.09) | 1.11 (1.13) | 1.14 (1.28) | 1.08 (1.18) | 1.11 (1.24) | 1.14 (1.31) |
| Dig.Met + Cys % | 0.86 | 0.89 | 0.91 | 0.86 | 0.89 | 0.91 | 0.86 | 0.89 | 0.91 | 0.86 | 0.89 | 0.91 |
| Dig.Thr % | 0.72 (0.76) | 0.74 (0.76) | 0.76 (0.78) | 0.72 (0.73) | 0.74 (0.81) | 0.76 (0.81) | 0.72 (0.77) | 0.74 (0.79) | 0.76 (0.85) | 0.72 (0.82) | 0.74 (0.83) | 0.76 (0.90) |
| Dig.Ile % | 0.76 (0.77) | 0.78 (0.79) | 0.80 (0.80) | 0.76 (0.74) | 0.78 (0.83) | 0.80 (0.83) | 0.76 (0.80) | 0.78 (0.81) | 0.80 (0.88) | 0.76 (0.82) | 0.78 (0.85) | 0.80 (0.91) |
| Dig.Leu % | 1.33 (1.35) | 1.37 (1.38) | 1.40 (1.39) | 1.33 (1.31) | 1.37 (1.45) | 1.40 (1.46) | 1.34 (1.40) | 1.37 (1.42) | 1.40 (1.52) | 1.34 (1.45) | 1.37 (1.48) | 1.41 (1.58) |
| Dig.Trp % | 0.23 | 0.24 | 0.24 | 0.23 | 0.24 | 0.24 | 0.23 | 0.24 | 0.24 | 0.23 | 0.24 | 0.24 |
| Dig.Arg % | 1.17 (1.25) | 1.20 (1.27) | 1.24 (1.27) | 1.17 (1.18) | 1.20 (1.27) | 1.24 (1.31) | 1.17 (1.24) | 1.20 (1.28) | 1.24 (1.29) | 1.17 (1.22) | 1.20 (1.25) | 1.24 (1.31) |
| Dig.His % | 0.46 (0.55) | 0.47 (0.55) | 0.48 (0.58) | 0.46 (0.53) | 0.47 (0.53) | 0.48 (0.57) | 0.46 (0.48) | 0.47 (0.53) | 0.48 (0.58) | 0.46 (0.52) | 0.47 (0.52) | 0.48 (0.55) |
| Dig.Val % | 0.85 (1.03) | 0.87 (1.03) | 0.89 (1.09) | 0.85 (1.00) | 0.87 (1.01) | 0.89 (1.07) | 0.85 (0.97) | 0.87 (1.01) | 0.89 (1.05) | 0.85 (0.96) | 0.87 (0.96) | 0.89 (1.02) |
| Crude Fat % | 5.72 (5.45) | 5.92 (5.90) | 6.13 (6.21) | 4.78 (4.55) | 4.97 (5.11) | 5.19 (5.32) | 3.84 (3.95) | 4.02 (3.94) | 4.23 (4.45) | 2.66 (3.11) | 2.91 (3.15) | 3.18 (3.45) |
| Crude Fiber % | 3.48 | 3.50 | 3.52 | 3.50 | 3.51 | 3.54 | 3.51 | 3.53 | 3.55 | 3.20 | 3.31 | 3.42 |
| Starch % | 44.2 (45.4) | 43.3 (44.1) | 42.3 (42.8) | 44.9 (45.7) | 44.1 (43.3) | 43.0 (43.7) | 45.7 (45.9) | 44.8 (45.4) | 43.8 (44.6) | 47.0 (46.4) | 46.0 (45.2) | 44.8 (45.7) |
| Phytate P % | 0.25 | 0.25 | 0.25 | 0.25 | 0.25 | 0.25 | 0.25 | 0.25 | 0.25 | 0.25 | 0.25 | 0.25 |
| Ca % | 0.75 (0.71) | 0.75 (0.68) | 0.75 (0.65) | 0.75 (0.68) | 0.75 (0.67) | 0.75 (0.72) | 0.75 (0.65) | 0.75 (0.67) | 0.75 (0.65) | 0.75 (0.66) | 0.75 (0.70) | 0.75 (0.64) |
| Avai P % | 0.38 | 0.38 | 0.38 | 0.38 | 0.38 | 0.38 | 0.38 | 0.38 | 0.38 | 0.38 | 0.38 | 0.38 |
| Total P % | 0.44 (0.50) | 0.44 (0.51) | 0.44 (0.51) | 0.44 (0.47) | 0.44 (0.49) | 0.44 (0.52) | 0.44 (0.48) | 0.44 (0.47) | 0.45 (0.50) | 0.44 (0.48) | 0.44 (0.52) | 0.45 (0.53) |
| Na % | 0.19 (0.15) | 0.19 (0.16) | 0.19 (0.14) | 0.19 (0.15) | 0.19 (0.16) | 0.19 (0.16) | 0.19 (0.15) | 0.19 (0.17) | 0.19 (0.15 | 0.19 (0.16) | 0.19 (0.17) | 0.19 (0.16) |
| Cl % | 0.22 | 0.22 | 0.22 | 0.22 | 0.22 | 0.22 | 0.22 | 0.22 | 0.22 | 0.22 | 0.22 | 0.22 |
| K % | 0.73 (0.78) | 0.75 (0.82) | 0.78 (0.80) | 0.73 (0.74) | 0.75 (0.81) | 0.78 (0.84) | 0.74 (0.80) | 0.75 (0.84) | 0.78 (0.84) | 0.73 (0.77) | 0.75 (0.83) | 0.78 (0.85) |
| Na+K-Cl meq/kg | 208 | 213 | 219 | 208 | 213 | 219 | 209 | 213 | 219 | 208 | 213 | 219 |

^1^CAA: control AA density, MAA: medium AA density (+3.0%), HAA: high AA density (+6.0%);

^2^ mg digestible Lys per 1000 kcal of ME

^3^ Digestible

**Table S4.** Calculated and measured nutrient content of the withdrawal diets (35-42 d). Values in parentheses are analyzed values and for amino acids represents total not digestible.

| Nutrients | Control ME (3150) | | | ME – 50 kcal (3100) | | | ME – 100 kcal (3050) | | | ME – 150 Kcal (3000) | | |
| --- | --- | --- | --- | --- | --- | --- | --- | --- | --- | --- | --- | --- |
|  | CAA^1^ | MAA | HAA | CAA | MAA | HAA | CAA | MAA | HAA | CAA | MAA | HAA |
| AME Kcal/kg | 3150 | 3150 | 3150 | 3100 | 3100 | 3100 | 3050 | 3050 | 3050 | 3000 | 3000 | 3000 |
| GE kcal/kg | 4145 | 4131 | 4145 | 4078 | 4083 | 4110 | 3979 | 3984 | 3985 | 3905 | 3923 | 3936 |
| Lys/ME ratio^2^ | 324 | 333 | 343 | 329 | 339 | 349 | 334 | 344 | 354 | 340 | 350 | 360 |
| Moisture % | 9.99 | 9.93 | 9.87 | 10.10 | 10.04 | 9.99 | 10.21 | 10.16 | 10.10 | 10.26 | 10.23 | 10.20 |
| Crude protein % | 19.9 (19.4) | 20.4 (19.9) | 20.9 (21.4) | 19.9 (20.3) | 20.4 (20.6) | 20.9 (20.8) | 19.9 (19.5) | 20.4 (19.9) | 20.9 (20.7) | 19.9 (19.6) | 20.4 (20.7) | 20.9 (21.1) |
| Dig.Lys^3^ % | 1.02 (1.03) | 1.05 (1.05) | 1.08 (1.13) | 1.02 (1.06) | 1.05 (1.07) | 1.08 (1.14) | 1.02 (1.03) | 1.05 (1.08) | 1.08 (1.11) | 1.02 (1.06) | 1.05 (1.06) | 1.08 (1.09) |
| Dig.Met + Cys % | 0.82 | 0.84 | 0.86 | 0.82 | 0.84 | 0.86 | 0.82 | 0.84 | 0.86 | 0.82 | 0.84 | 0.86 |
| Dig.Thr % | 0.68 (0.71) | 0.70 (0.73) | 0.72 (0.78) | 0.68 (0.72) | 0.70 (0.74) | 0.72 (0.78) | 0.68 (0.71) | 0.70 (0.71) | 0.72 (0.72) | 0.68 (0.72) | 0.70 (0.73) | 0.72 (0.74) |
| Dig.Ile % | 0.70 (0.72) | 0.73 (0.75) | 0.75 (0.81) | 0.70 (0.73) | 0.73 (0.76) | 0.75 (0.80) | 0.70 (0.73) | 0.73 (0.73) | 0.75 (0.74) | 0.71 (0.71) | 0.73 (0.75) | 0.75 (0.77) |
| Dig.Leu % | 1.25 (1.27) | 1.29 (1.32) | 1.32 (1.42) | 1.25 (1.28) | 1.29 (1.34) | 1.32 (1.40) | 1.25 (1.28) | 1.29 (1.28) | 1.33 (1.36) | 1.25 (1.26) | 1.29 (1.32 | 1.33 (1.36) |
| Dig.Trp % | 0.22 | 0.22 | 0.23 | 0.22 | 0.22 | 0.23 | 0.22 | 0.22 | 0.23 | 0.22 | 0.22 | 0.23 |
| Dig.Arg % | 1.12 (1.16) | 1.16 (1.17) | 1.19 (1.25) | 1.12 (1.09) | 1.16 (1.21) | 1.19 (1.23) | 1.12 (1.16) | 1.16 (1.18) | 1.19 (1.22) | 1.12 (1.14) | 1.16 (1.21) | 1.19 (1.25) |
| Dig.His % | 0.43 (0.46) | 0.44 (0.47) | 0.46 (0.51) | 0.43 (0.47) | 0.44 (0.48) | 0.46 (0.50) | 0.43 (0.46) | 0.44 (0.46) | 0.46 (0.47) | 0.43 (0.46) | 0.45 (0.47) | 0.46 (0.49) |
| Dig.Val % | 0.80 (0.85) | 0.82 (0.87) | 0.84 (0.93) | 0.80 (0.85) | 0.82 (0.88) | 0.84 (0.92) | 0.80 (0.85) | 0.82 (0.85) | 0.84 (0.88) | 0.80 (0.83) | 0.82 (0.87) | 0.84 (0.89) |
| Crude Fat % | 6.38 (6.21) | 6.58 (6.36) | 6.79 (6.60) | 5.43 (5.10) | 5.63 (5.32) | 5.85 (5.62) | 4.49 (4.15) | 4.69 (4.51) | 4.90 (4.57) | 3.37 (3.15) | 3.65 (3.54) | 3.92 (3.59) |
| Crude Fiber % | 3.62 | 3.64 | 3.66 | 3.63 | 3.66 | 3.68 | 3.65 | 3.67 | 3.69 | 3.44 | 3.55 | 3.67 |
| Starch % | 45.2 (46.4) | 44.2 (45.7) | 43.2 (44.7) | 46.0 (46.5) | 45.0 (44.7) | 44.0 (44.7) | 46.7 (45.3) | 45.8 (46.6) | 44.8 (45.5) | 47.9 (47.2) | 46.7 (46.8) | 45.6 (46.5) |
| Phytate P % | 0.25 | 0.25 | 0.25 | 0.25 | 0.25 | 0.25 | 0.25 | 0.25 | 0.25 | 0.25 | 0.25 | 0.25 |
| Ca % | 0.70 (0.62) | 0.70 (0.58) | 0.70 (0.60) | 0.70 (0.63) | 0.70 (0.57) | 0.70 (0.61) | 0.70 (0.62) | 0.70 (0.59) | 0.70 (0.61) | 0.70 (0.57) | 0.70 (0.57) | 0.70 (0.59) |
| Avai P % | 0.35 | 0.35 | 0.35 | 0.35 | 0.35 | 0.35 | 0.35 | 0.35 | 0.36 | 0.35 | 0.35 | 0.36 |
| Total P % | 0.41 (0.47) | 0.41 (0.44) | 0.42 (0.45) | 0.41 (0.45) | 0.41 (0.47) | 0.42 (0.46) | 0.41 (0.48) | 0.41 (0.45) | 0.42 (0.47) | 0.41 (0.45) | 0.41 (0.44) | 0.42 (0.48) |
| Na % | 0.19 (0.15) | 0.19 (0.14) | 0.19 (0.16) | 0.19 (0.15) | 0.19 (0.15) | 0.19 (0.16) | 0.19 (0.17) | 0.19 (0.16) | 0.19 (0.16) | 0.19 (0.15) | 0.19 (0.16) | 0.19 (0.16) |
| Cl % | 0.22 | 0.22 | 0.22 | 0.22 | 0.22 | 0.22 | 0.22 | 0.22 | 0.22 | 0.22 | 0.22 | 0.22 |
| K % | 0.69 (0.74) | 0.71 (0.73) | 0.73 (0.78) | 0.69 (0.73) | 0.71 (0.77) | 0.73 (0.77) | 0.69 (0.71) | 0.71 (0.75) | 0.73 (0.78) | 0.69 (0.75) | 0.71 (0.75) | 0.73 (0.76) |
| Na+K-Cl meq/kg | 197 | 202 | 208 | 197 | 202 | 208 | 197 | 202 | 208 | 196 | 202 | 208 |

^1^CAA: control AA density, MAA: medium AA density (+3.0%), HAA: high AA density (+6.0%);

^2^ mg digestible Lys per 1000 kcal of ME

^3^ Digestible
